# Cardiometabolic screening, elevated results and clinical follow-up actions in Māori and non-Māori with psychosis in Canterbury, Aotearoa New Zealand

**DOI:** 10.1177/00048674261461979

**Published:** 2026-07-04

**Authors:** Nathan Monk, Emily Barrett, Julie Fitzjohn, Mau Te Rangimarie Clark, Melissa Kerdemelidis, Ruth Cunningham, Andre McLachlan, Waikaremoana Waitoki, Richard Porter, Cameron Lacey

**Affiliations:** 1 Department of Psychological Medicine, University of Otago, Christchurch, New Zealand; 2 Department of Māori/Indigenous Health Innovation, University of Otago, Christchurch, New Zealand; 3 Specialist Mental Health Service, Health New Zealand – Canterbury (Te Whatu Ora – Waitaha), Christchurch, New Zealand; 4 Population Health Gain, Service Improvement and Innovation, Health New Zealand – Canterbury (Te Whatu Ora – Waitaha), Christchurch, New Zealand; 5 Department of Public Health, University of Otago, Wellington, New Zealand; 6 Centre for Health and Social Practice, Waikato Institute of Technology, Hamilton, New Zealand; 7 Faculty of Māori and Indigenous Studies, The University of Waikato, Hamilton, New Zealand; 8 Elimbias Health, Christchurch, New Zealand

**Keywords:** Psychosis, Māori health, cardiometabolic risk, health inequity, antipsychotic medication, HbA1c, lipids

## Abstract

**Objective:**

Estimate and compare rates of cardiometabolic blood screening, elevated results and clinical follow-up actions for Māori and non-Māori mental health service users with psychosis in Canterbury, Aotearoa New Zealand.

**Methods:**

A retrospective cohort study (*N* = 3840) with dynamic entry was conducted with people aged 18–64 years with a psychosis diagnosis in Canterbury Specialist Mental Health Service (1 August 2012 to 31 December 2019). Linked administrative data captured lipid and HbA1c screening within 1 year of study entry, elevated results (triglycerides > 2.2 mmol/L; total cholesterol:high-density lipoprotein cholesterol > 5; HbA1c > 40 mmol/mol) from individual study entry to 31 December 2023, and clinical actions (repeat test; statin/metformin dispensing) within 1 year following an elevated result.

**Results:**

887/3840 (23%) of Canterbury Specialist Mental Health Service users with psychosis were Māori. First-year screening rates were modest (31–44%), similar by ethnicity and broadly higher among clozapine/olanzapine users. Māori had higher odds of both elevated lipids and HbA1c. About one-third of elevated results were followed up with re-testing within 1 year. Rates of follow-up testing and medication dispensing were broadly similar for Māori and non-Māori, except Māori were more likely to receive repeat HbA1c testing after an elevated result (odds ratio = 1.71 [1.16, 2.50]).

**Conclusions:**

Findings highlight large gaps in cardiometabolic screening and follow-up actions among Māori and non-Māori Specialist Mental Health Service users with psychosis. Māori presented with psychosis 4 years younger, had significantly higher rates of elevated cardiometabolic risk markers and were more likely to receive HbA1c re-testing following an elevated result.

People with psychosis experience a higher risk of many physical health problems and associated mortality (Correll et al., 2017; de Hert et al., 2011; Firth et al., 2019). Greater cardiovascular and metabolic risks are associated with psychosis, particularly in psychosis treated with second-generation antipsychotic medications (Solmi et al., 2017).

Guidelines for the clinical management of psychosis (Galletly et al., 2016; McCutcheon et al., 2025), Aotearoa New Zealand (NZ) national cardiovascular disease (CVD) risk assessment guidelines (Ministry of Health, 2018), and the New Zealand Formulary (2026) recommend at least annual physical health monitoring of people with psychosis, including blood testing. In particular, lipids and blood sugar (glycated haemoglobin [HbA1c]) and/or fasting blood glucose) are important blood markers for CVD and diabetes risk. The New Zealand Formulary (2026) recommends that people taking clozapine or olanzapine should receive blood testing regularly, including an annual lipid profile and fasting blood glucose every 4–6months. In sum, all people in this study should be receiving at least an annual cardiometabolic blood screening.

In NZ, people with psychosis may be treated in primary or specialist health services, including multidisciplinary early intervention psychosis services, which are present in 11 of 20 districts nationwide (Grattan et al., 2025). In Canterbury, NZ (the setting of this study), the local early intervention psychosis service delivers care for up to 2 years to people aged 18–28 years experiencing a first episode of psychosis. The responsibility for physical health monitoring (including ordering recommended blood tests) of people with psychosis in the broader population is thus spread across both primary and specialist services. However, younger people experiencing first-episode psychosis (FEP) will more frequently be under the care of specialist early intervention psychosis services, compared to older people, for whom clinical management is undertaken by generic Specialist Mental Health Services (SMHSs) with transfer to primary care where possible.

Despite extensive and growing data on inequities, NZ psychosis research is yet to meaningfully respond to formal recommendations for *Tiriti o Waitangi*-based Māori health advancement (see, in particular, the Whakamaua Māori Health Action Plan and recommendations from the Hauora Report; Ministry of Health, 2020; Waitangi Tribunal, 2023). However, as a basic *Tiriti*-aligned stratagem for reporting quantitative data, Māori and non-Māori statistics are typically presented independently in quantitative psychosis research in NZ (e.g. Cunningham et al., 2025; Manuel et al., 2023; Petrović-van der Deen et al., 2020). This approach ensures that research is sensitive to Māori health equity, can contextualise the sources of inequity and can inform equitable service improvement. This is particularly important in psychosis healthcare research, as Māori are diagnosed with psychosis twice as frequently and experience higher psychosis-related health risks (Cunningham et al., 2025; Monk et al., 2024b; Petrović-van der Deen et al., 2020).

This study builds on previous work which identified gaps in cardiometabolic blood screening receipt among NZ SMHS users with psychosis (Monk et al., 2024a). In this study, we use regional SMHS and healthcare data from the Canterbury province in NZ’s South Island. There are no previously published data on cardiometabolic testing rates in Canterbury people with psychosis. Unlike the national administrative data used in previous research (Monk et al., 2024a), these regional data contain the results of blood tests, which enable identification of elevated lipid and HbA1c levels, and subsequent clinical actions. As clozapine and olanzapine use are associated with especially high cardiometabolic risk (Komossa et al., 2010; Wagner et al., 2021), we also present each analysis for both (a) all participants and (b) a subset of only participants dispensed clozapine or olanzapine. The aims of this study are as follows:

1. Describe rates of lipid and HbA1c screening completion in the first year following SMHS contact.
2. Describe rates of elevated lipid and HbA1c test results at any time during the study.
3. Describe rates of follow-up re-testing and dispensing of physical health medications (statins and metformin) within 1 year following elevated lipid and HbA1c test results.
4. For 1–3, compare Māori and non-Māori.

## Method

### Ethics

The authors assert that all procedures contributing to this work comply with the ethical standards of the relevant national and institutional committees on human experimentation and with the Helsinki Declaration of 1975, as revised in 2008. All procedures involving human subjects/patients were approved by the University of Otago Ethics Committee, reference number HD22/128. Participant consent is not required to analyse de-identified administrative data (Te Whatu Ora/Health New Zealand, 2023).

#### Participants

Patients with a psychosis diagnosis (see Table 1 for included diagnoses) between 1 August 2012 and 31 December 2019 were identified from Canterbury SMHS data held by Health New Zealand – Canterbury (Te Whatu Ora – Waitaha). Data were originally extracted in two cohorts: *rangatahi* (young people) aged 18–24 years and *pakeke* (adults) aged 25–64 years. As *rangatahi* are often under dedicated early psychosis care, it was planned to analyse data separately for *rangatahi* and *pakeke*. However, after stratifying the cohorts by both ethnicity and instances of elevated test results, the *rangatahi* cohort was too small to adequately power statistical analysis. Thus, we pooled the two cohorts for all analyses and report a sensitivity analysis of the effect of participant sub-cohort on screening receipt. The final combined cohort thus represents all patients aged 18–64 years with a psychosis diagnosis recorded in Canterbury SMHS between 1 August 2012 and 31 December 2019.

**Table 1.** Age, gender and psychosis diagnoses of Māori and non-Māori Specialist Mental Health Service (SMHS) users with psychosis in Canterbury from 1 August 2012 to 31 December 2019.

	Māori (*N* = 887)		Non-Māori (*N* = 2953)	
	*n*	%	*n*	%
*Sub-cohort*				
Rangatahi (18–24 years)	230	25.9%	499	16.9%
Pakeke (25–64 years)	657	74.1%	2454	83.1%
*Overall mean age in years (SD)*	34.7	(12.0)	39.1	(12.8)
*Gender*				
Male	561	63.2%	1680	56.9%
Female	336	37.9%	1283	43.4%
Another gender	0	0.0%	2	0.1%
*Median (IQR) follow-up years* ^a^	8	(6–10)	8	(6–10)
*Psychosis diagnoses* ^b,c^				
Schizophrenia	254	28.6%	906	30.7%
Bipolar type I	172	19.4%	1012	34.3%
Schizoaffective	127	14.3%	462	15.6%
Substance-induced	62	7.0%	198	6.7%
Depression with psychosis	39	4.4%	325	11.0%
Delusional	19	2.1%	194	6.6%
Brief	4	0.5%	33	1.1%
Organic	8	0.9%	28	0.9%
Psychosis not otherwise specified (NOS)	354	39.9%	1266	42.9%

a Time in years from individual study entry (psychosis diagnosis in SMHS) and end of full observation window (31 December 2023).

b All psychosis diagnoses recorded in SMHS for each person included in the study (thus percentages sum to >100).

c DSM-IV and ICD-10 codes are given in Table S1 in Supplementary Materials.

From the SMHS data, all recorded psychosis diagnoses for each person were obtained for descriptive purposes. Data on the type and frequency of mental health service contacts were unavailable. Each person’s first SMHS contact between 1 August 2012 and 31 December 2019 was taken as their study index date for follow-up analysis.

#### Design

The study is a retrospective cohort design with dynamic individual entry based on SMHS service contact with psychosis (see Figure 1). Patient data were linked between sources via a hashed (de-identified) National Health Index (NHI) number.

**Figure 1.**
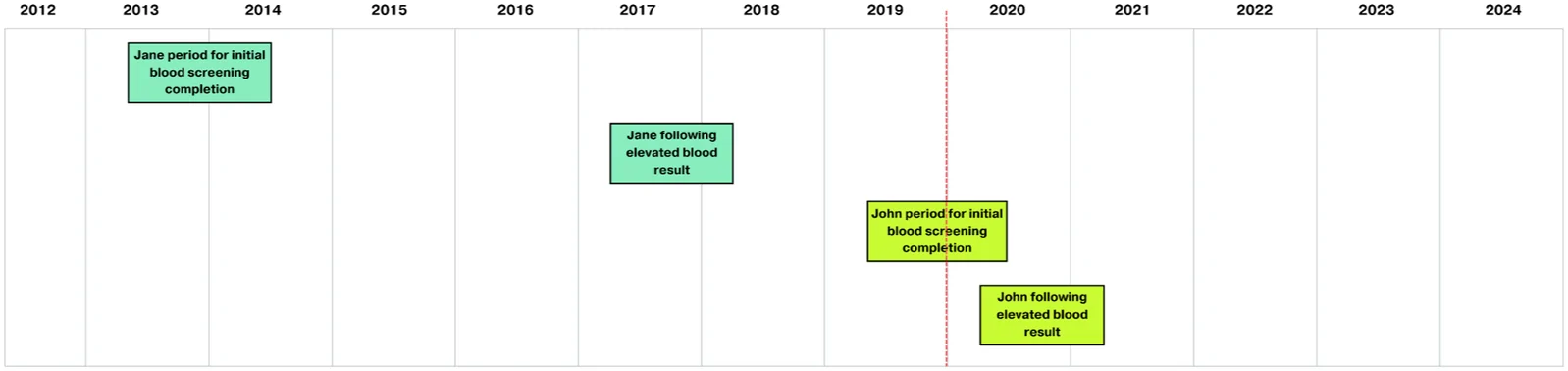
The retrospective cohort study design with dynamic entry, visualised using two hypothetical participants. Jane entered the study with a psychosis diagnosis in SMHS in June 2013 and later had an elevated blood result in April 2017. John entered the study with a psychosis diagnosis in SMHS in June 2019 and had an elevated blood result in April 2020. The cut-off date for study inclusion (indicated by a dotted red line) is 31 December 2019. The initial screening periods are 14 months, including a 2-month lookback period to account for inconsistencies in data processing between SMHS and laboratory data. The period following elevated blood results is 12 months with no lookback period.

#### Measures

##### Ethnicity

Prioritised patient ethnicity (Māori ethnicity prioritised over others if multiple ethnicities recorded) was based on self-identified ethnicity reported in SMHS records, consistent with national ethnicity data handling and reporting protocols (Health Information Standards Organisation, 2017). All analyses compare Māori and non-Māori.

##### Healthcare data

Laboratory and medication dispensing data were extracted from the Health New Zealand – Canterbury (Te Whatu Ora – Waitaha) data warehouse. Two categories of blood screening tests were extracted from laboratory data: lipids (total cholesterol [TC], high-density lipoprotein (HDL) cholesterol, low-density lipoprotein cholesterol, triglycerides and TC:HDL-C) and HbA1c. Records of dispensing events for clozapine, olanzapine, statins and metformin were retrieved from both community and inpatient sources.

#### Analysis

All data cleaning and analyses were performed in R (version 4.4.2, R Institute, Vienna, Austria). Gender, psychosis diagnoses and age are presented descriptively for Māori and non-Māori (Table 1). Bivariate logistic regression was used to compare Māori and non-Māori on all outcomes. Visual inspection of log-odds between age and screening outcomes showed clear non-linearity. To preserve interpretability, we present only bivariate (not age-adjusted) logistic regression models. We report a sensitivity analysis of the effect of the original sub-cohort (*rangatahi* aged 18–24 years vs *pakeke* aged 25–64 years) on the odds of screening receipt. Results for all main models adjusted for sub-cohort are presented in Supplementary Tables S2–S4 (these adjusted results are not substantively different from the main results; one minor difference is reported in Results).

First, we estimated rates of cardiometabolic screening (lipids and HbA1c) receipt within 1 year of their study index date (first SMHS contact during the study period). To account for different data reporting processes between SMHS and the laboratory data set, blood tests recorded within 2 months prior to a person’s index date were also considered completed. Second, we estimated rates of elevated lipid and HbA1c test results at any time following study entry until 31 December 2023. Elevated results were defined as follows: triglycerides (lipids) > 2.2 mmol/L, TC:HDL cholesterol (lipids) > 5 and HbA1c > 40 mmol/mol (pre-diabetic). Third, we estimated rates of clinical actions within 1 year following an elevated test result. For each type of elevated result, two subsequent actions were identified: re-test within 1 year (same test repeated) and medication dispensed within 1 year (statins for elevated lipids; metformin for elevated HbA1c).

In identifying people who were dispensed olanzapine or clozapine for sub-analyses, two approaches were used in different analyses. For the analysis of first-year screening receipt, people were categorised as clozapine/olanzapine users if they received either medication at least once during the same period. For any-time elevated results and follow-up, people were categorised as clozapine/olanzapine users if they were dispensed clozapine or olanzapine at any time following their index date.

## Results

A total of 3840 people with psychosis diagnoses were identified from SMHS records between 1 August 2012 and 31 December 2019, of whom 887 (23.1%) were Māori (Table 1). Most individuals were *pakeke* aged 25–64 years, with Māori being 4.4 years younger on average than non-Māori (mean age 34.7 vs 39.1 years). Most participants were men, with a larger gender imbalance in Māori. Schizophrenia was the most common psychosis diagnosis overall, while affective psychoses (bipolar type I and depression with psychosis) were notably more frequent in non-Māori than Māori. Median follow-up time in years was the same for Māori and non-Māori (median = 8, interquartile range [IQR] = 6–10).

### Blood screening within one year of service contact (Table 2)

Overall, less than half of the cohort had lipid screening (43–44%) and less than one-third had HbA1c screening (31–33%) in the year following first-identified diagnosis or service contact. Screening rates for both lipids and HbA1c during the first year were similar for Māori and non-Māori. Among clozapine/olanzapine users only, screening rates appeared broadly higher than for the general cohort: about half (51–55%) of clozapine/olanzapine users received lipid screening and around 4-in-10 received HbA1c screening (38–44%). These rates were also similar between Māori and non-Māori; however, Māori clozapine/olanzapine users had significantly higher odds of receiving HbA1c screening compared to non-Māori (OR = 1.29 [1.04, 1.60]).

**Table 2.** Rates of lipid and HbA1c screening completion for Māori and non-Māori within 1 year of first Specialist Mental Health Service (SMHS) use in Canterbury between 1 August 2012 and 31 December 2019.

	Māori (*N* = 887)		Non-Māori (*N* = 2953)		OR (95% CI)
*All service users*	*n*	%	*n*	%	
Lipids	391	44.1%	1276	43.2%	1.04 (0.89, 1.20)
HbA1c	292	32.9%	918	31.1%	1.09 (0.93, 1.28)
	Māori (*N* = 440)		Non-Māori (*N* = 1434)		OR (95% CI)
*Olanzapine/clozapine users only*	*n*	%	*n*	%	
Lipids	240	54.5%	734	51.2%	1.14 (0.92, 1.42)
HbA1c	193	43.9%	541	37.7%	1.29* (1.04, 1.60)

Both blood tests and antipsychotic use were recorded if they occurred within 2 months prior to or 1 year after each person’s study index date (so the follow-up period ended on 31 December 2020).

* *p* < .05.

### Sensitivity analysis

As Māori were more frequently from the younger *rangatahi* sub-cohort (26% vs 17% of non-Māori), and we theorised a sub-cohort effect due to the different healthcare settings (early intervention service vs general SMHS or primary care), we performed sensitivity analyses to estimate the effect of original sub-cohort (*rangatahi* aged 18–24 years vs *pakeke* aged 25–64 years) on screening receipt. *Rangatahi* were more likely than *pakeke* to receive lipid screening (OR = 1.18 [1.01, 1.39]). This remained significant after adjusting for Māori/non-Māori ethnicity (OR = 1.18 [1.00, 1.39]). There was no significant difference in odds of HbA1c screening receipt (OR = 1.06 [0.89, 1.26]). All main models were re-estimated with adjustment for sub-cohort and presented in Supplementary Tables S2–S4. One marginally significant model from the main paper became insignificant after sub-cohort adjustment: there was no difference in adjusted odds of Māori and non-Māori clozapine/olanzapine users receiving statins after elevated triglycerides (Adjusted OR = 0.75 [0.48, 1.15]). All other significant models remained, and effect estimates were not substantively different.

### Elevated lipid and HbA1c results across follow-up (Table 3)

For both Māori and non-Māori, the median follow-up time after study entry was 8 years (IQR = 6–10). Between study entry and 31 December 2023, 86% of individuals had at least one lipid or HbA1c test recorded, with little difference between Māori (85%) and non-Māori (86%). Māori were more likely to have elevated triglycerides (31% vs 23%; OR = 1.49 [1.26, 1.76]), elevated TC:HDL (23% vs 18%; OR = 1.32 [1.10, 1.58]) and elevated HbA1c (18% vs 12%; OR = 1.62 [1.32, 1.99]) at any point in follow-up.

**Table 3.** Number of Māori and non-Māori Canterbury Specialist Mental Health Service (SMHS) users with an elevated lipid and HbA1c blood screening result between study entry and 31 December 2023.

		Māori (*N* = 887)	Non-Māori (*N* = 2953)		OR (95% CI)
*All service users*	*n*	%	*n*	%	
Triglycerides > 2.2 mmol/L (lipids)	274	30.9%	681	23.1%	1.49*** (1.26, 1.76)
TC:HDL-C > 5 (lipids)	202	22.8%	539	18.3%	1.32** (1.10, 1.58)
HbA1c > 40 mmol/mol	156	17.6%	343	11.6%	1.62*** (1.32, 1.99)
		Māori (*N* = 561)	Non-Māori (*N* = 1815)		OR (95% CI)
*Any-time Olanzapine/clozapine users only*	*n*	%	*n*	%	
Triglycerides > 2.2 mmol/L (lipids)	223	39.8%	512	28.2%	1.68*** (1.38, 2.04)
TC:HDL-C > 5 (lipids)	158	28.2%	393	21.7%	1.42** (1.14, 1.76)
HbA1c > 40 mmol/mol	112	20.0%	242	13.3%	1.62*** (1.26, 2.07)

This analysis includes all blood tests taken after each person’s study index date (earliest possible date 1 August 2012, up to 31 December 2023).

Antipsychotic use for this analysis is recorded any time following study entry (and up to 2 months prior), so antipsychotic use is not necessarily contemporaneous with the elevated result.

** *p* < .01; ***p < .001.

Overall, 63% of Māori and 61% of non-Māori were dispensed clozapine or olanzapine at some point during follow-up. Among this group, elevated results were more common, and comparable differences remained between Māori and non-Māori. Māori clozapine/olanzapine users were more likely to have elevated triglycerides (40% vs 28%; OR = 1.68 [1.38, 2.04]), elevated TC:HDL-C (28% vs 22%; OR = 1.42 [1.14, 1.76]) and elevated HbA1c (20% vs 13%; OR = 1.62 [1.26, 2.07]). It is notable that one-in-five Māori and approximately one-in-eight non-Māori dispensed clozapine or olanzapine met the clinical threshold for pre-diabetes (HbA1c > 40).

### Clinical actions within 1 year following an elevated result (Table 4)

Measured actions after an elevated blood result were generally limited. Rates of lipids re-testing within 1 year were modest (37–38%) and did not differ significantly between Māori and non-Māori. However, Māori were significantly more likely to receive a repeat HbA1c test within 1 year after an elevated result (52% vs 39%; OR = 1.71 [1.16, 2.50]). This difference was not explained by higher initial HbA1c levels, which were similar between groups (mean results among elevated HbA1c tests: Māori 51.4 mmol/mol, *SD* = 15.0; non-Māori 51.0 mmol/mol, *SD* = 13.4). Re-testing rates were similar when looking only at people dispensed clozapine/olanzapine. In this sub-group, Māori were again more likely to have HbA1c re-tested following an elevated result (60% vs 43%; OR = 2.01 [1.28, 3.18]).

**Table 4.** Rate of follow-up action within 1 year following elevated lipid and/or HbA1c blood test results, Māori and non-Māori Canterbury Specialist Mental Health Service (SMHS) users with psychosis.

	Elevated result (*n*)		% (*n*) re-tested following an elevated blood result			% (*n*) dispensed statin (lipids)/metformin (HbA1c) following elevated blood result		
	Māori (*N* = 887)	Non-Māori (*N* = 2953)	Māori	Non-Māori	OR (95% CI)	Māori	Non-Māori	OR (95% CI)
*All service users*								
Triglycerides > 2.2 mmol/L (lipids)	274	681	38.0% (104)	37.0% (252)	1.04 (0.78, 1.39)	16.1% (44)	20.4% (139)	0.75 (0.51, 1.07)
TC:HDL-C > 5 (lipids)	202	539	37.6% (76)	38.4% (207)	0.97 (0.69, 1.35)	13.4% (27)	16.0% (86)	0.67 (0.39, 1.13)
HbA1c > 40 mmol/mol	156	343	51.9% (81)	38.8% (133)	1.71** (1.16, 2.50)	42.9% (67)	39.7% (136)	1.15 (0.78, 1.68)
	Māori (*N* = 561)	Non-Māori (*N* = 1815)	Māori	Non-Māori		Māori	Non-Māori	
*Any-time olanzapine/clozapine users only*								
Triglycerides > 2.2 mmol/L (lipids)	223	512	36.8% (86)	37.9% (204)	0.95 (0.69, 1.31)	14.5% (32)	19.1% (105)	0.65* (0.42, 0.99)
TC:HDL-C > 5 (lipids)	158	393	38.0% (60)	41.7% (164)	0.85 (0.58, 1.25)	14.3% (22)	16.8% (66)	0.62 (0.33, 1.10)
HbA1c > 40 mmol/mol	112	242	59.8% (67)	42.6% (103)	2.01** (1.28, 3.18)	44.6% (50)	37.6% (91)	1.39 (0.85, 2.11)

All follow-up actions completed if recorded within 1 year of initial elevated blood result.

Follow-up percentages are the percentage of people with the corresponding elevated blood result.

Antipsychotic use for this analysis is recorded any time following study entry (and up to 2 months prior).

* *p* < .05; **p < .01.

Following an elevated lipid result, statin dispensing was uncommon for both Māori and non-Māori (13–20%). Metformin dispensing following elevated HbA1c was more common (40–43%). Dispensing rates for neither of these medications were significantly different between Māori and non-Māori. Among people dispensed clozapine/olanzapine at any time, patterns were similar, with modest dispensing rates for statins (14–19%). However, among this sub-group, non-Māori had marginally significantly higher odds of receiving statins following elevated triglycerides (OR = 0.65 [0.42, 0.99]; note this became insignificant after adjusting for sub-cohort, see Supplementary Table S4). Rates of metformin dispensing were again higher (38–45%), again with no significant difference between Māori and non-Māori.

## Discussion

We estimated rates of cardiometabolic blood screening, elevated blood results and subsequent clinical actions for Māori and non-Māori SMHS users with psychosis diagnoses in the Canterbury province of NZ. Consistent with national-level data (Petrović-van der Deen et al., 2020), Māori were over-represented approximately twofold in this psychosis cohort: 23.1% of this age 18–64 years cohort were Māori, whereas around 10% of the all-ages Canterbury general population are Māori (Statistics New Zealand, 2025). Māori were also 4 years younger than non-Māori in this Canterbury psychosis cohort, consistent with nationwide SMHS data (Monk et al., 2024a).

Results indicate that lipid and HbA1c testing are typically not occurring annually as recommended (Galletly et al., 2016; McCutcheon et al., 2025; Ministry of Health, 2018). Rates were generally higher among people dispensed clozapine or olanzapine (38–55%), suggesting some clinical action being taken to monitor increased cardiometabolic risk associated with these medications (Komossa et al., 2010; Wagner et al., 2021). However, overall, it appears that best practice cardiometabolic blood monitoring is occurring for fewer than half of people with psychosis in Canterbury, NZ. Moreover, only about one-third of people with elevated lipid or HbA1c test results were re-tested within 1 year. So monitoring guidelines are typically not being met in Canterbury for even those with the highest cardiometabolic risk (i.e. people with elevated blood markers).

Despite being 4 years younger on average, Māori had significantly higher odds (all *p* < .01) of all elevated results. Among people dispensed clozapine or olanzapine, one-in-five Māori met the threshold for at least pre-diabetes (HbA1c > 40 mmol/mol), compared with about one-in-eight non-Māori. This echoes previous national evidence of heightened diabetes risk among Māori experiencing psychosis (Monk et al., 2024a, 2024b).

Relatively few people with elevated lipids/HbA1c were dispensed statins/metformin. While these rates appear low, we note that statin/metformin prescriptions will not be clinically indicated by elevated lipid/HbA1c results alone. The decision to prescribe these medications depends for a wider range of factors. For instance, to inform statin prescribing, Canterbury clinicians typically use a CVD risk assessment tool which includes factors such as personal demographics, socioeconomic status, clinical history, smoking status, blood pressure and body mass index, as well as blood test results (Te Whatu Ora/Health New Zealand, 2025). The present analysis informs only on the use of these medications among people with higher cardiometabolic risk as per blood test results alone, so should not be read as receipt of best practice, which is a personalised, multifactorial calculation. However, very recent RANZCP guidelines recommend adjunct metformin for all people starting olanzapine or clozapine (Suetani et al., 2026). We found only modest rates (38–45%) of metformin dispensing among any-time olanzapine/clozapine users following a pre-diabetic or diabetic HbA1c result. This suggests that improvement is likely needed in metformin prescribing.

Few differences in cardiometabolic monitoring were observed between Māori and non-Māori. The common significant difference was that HbA1c monitoring is more responsive in Māori, particularly among clozapine/olanzapine users. This may indicate that equity is being considered in clinical decision-making for diabetes risk monitoring, as Māori with psychosis are around 1.5 times more likely to be hospitalised with diabetes than non-Māori (Monk et al., 2024a, 2024b).

In previous national data, we found that Māori were less likely to receive lipid and HbA1c screening than non-Māori over a 2-year period (Monk et al., 2024a). In the present regional data, Māori received at least equivalent rates of lipid and HbA1c screening. Sensitivity analyses showed that the greater representation of Māori in the younger *rangatahi* sub-cohort partly explains more responsive lipid screening. Many *rangatahi* with psychosis in Canterbury will be under the multidisciplinary care of the local early intervention psychosis service. From present evidence, it cautiously appears that the local early intervention psychosis service may be making a small but positive impact on overall cardiometabolic testing rates.

While we found few differences in the monitoring received by Māori and non-Māori, the significant overall gaps will lead to more undetected cardiometabolic risk in Māori. The present data show that cardiometabolic risk markers are all significantly more common in Māori (all *p* < .01) despite being 4 years younger, and previous studies show higher rates of cardiometabolic disease and mortality for Māori (Monk et al., 2024a, 2024b). In improving these statistics specifically for Māori, work is needed to embed culturally safe Kaupapa Māori models of care, such as the Meihana Model and Hui Process (Lacey et al., 2011; Pitama et al., 2014). This is a noted gap across NZ early psychosis services (Grattan et al., 2025). We recently conducted focus groups with members of the health workforce in Canterbury and Waikato (NZ) working with young Māori experiencing psychosis. One priority theme that consistently emerged was the need to increase the number of Māori in the health workforce (Clark et al., 2025), consistent with previous qualitative research in this area (Haitana et al., 2022a, 2022b, 2023).

However, we emphasise that large gaps in cardiometabolic testing for both Māori and non-Māori indicate that improving cultural safety alone is unlikely to cause significant improvement. These results suggest systemic accessibility issues which are likely to extend beyond quality and safety of face-to-face care. As an initiative aimed at improving this, Canterbury SMHS have identified a need to train more staff to take blood during appointments, which will help mitigate inaccessibility caused by sending patients to a lab for blood testing (personal communication between authors J.F. and N.M., 6 November 2025).

Internationally, there is a notable lack of evidence on the physical health and monitoring of Indigenous Peoples experiencing psychosis. However, Australia has a growing body of evidence on the epidemiology of Aboriginal and Torres Strait Islanders experiencing psychosis (Charlson et al., 2021; Gynther et al., 2019; Hunter et al., 2011). In addition to the local implications we outline, we also hope our findings serve as a basis to develop similar research which centres Indigenous Peoples with psychosis. While these are small population groups, the combination of psychosis with socioeconomic realities of colonised Indigenous Peoples renders these groups very high risk for preventable physical health conditions (see: Curtis et al., 2023). Nations subject to the United Nations Declaration on the Rights of Indigenous People are guided to act on this (United Nations (General Assembly), 2007: Articles 21, 23–24).

Key strengths of this study include the use of multiple sources of linked data: SMHS, laboratory processing and medication dispensing data spanning over a decade. This provided a regionally representative sample of Māori and non-Māori experiencing psychosis, and identification of important healthcare measures. The *Tiriti*-based analysis design places Māori and non-Māori on equal footing as Treaty partners. This upholds Māori sovereignty, allowing Māori statistics to be reported and contextualised, in line Indigenous guidelines for reporting in health research (Huria et al., 2019).

Several limitations should be noted. These data cover only a limited selection of healthcare screening and monitoring events. Primary care data were not available, meaning many possible clinically relevant health screening measures and follow-up actions which occur in primary care settings (e.g. smoking status, CVD risk assessment, weight/BMI and blood pressure taken, and lifestyle advice/interventions) are not captured. Dispensing data indicate prescriptions filled but not adherence or review. Our definition of olanzapine/clozapine users for Tables 3 and 4 (any-time users during follow-up period) meant that olanzapine/clozapine use did not have to be contemporaneous with the elevated result. Last, it has been reported that NZ administrative data sources tend to undercount Māori, nationally by as much as 16% in health data, so some Māori are likely erroneously classified as non-Māori in these data (Harris et al., 2022).

Presently, NZ does not have the data infrastructure to repeat and expand this work at the national level. At the local level in Canterbury, it should be explored how these findings could be expanded to capture aspects of psychosis healthcare which are managed in primary care alongside local Primary Health Organisations. In addition to further research on service use, further longitudinal research is needed on cardiometabolic risk monitoring and reduction following elevated results, as risk detection does not guarantee risk reduction. Effective interventions are also needed.

## Conclusion

These findings highlight large gaps in recommended cardiometabolic blood monitoring for Māori and non-Māori experiencing psychosis in Canterbury, NZ. Māori presented with psychosis 4 years younger, had significantly higher rates of elevated cardiometabolic risk markers and were more likely to receive HbA1c re-testing following an elevated result. We discuss critical areas for improvement in rates of screening and following up elevated results, which must be guided by *Te Tiriti* obligations.

## Supplemental Material

sj-docx-1-anp-10.1177_00048674261461979 – Supplemental material for Cardiometabolic screening, elevated results and clinical follow-up actions in Ma-ori and non-Ma-ori with psychosis in Canterbury, Aotearoa New Zealand Supplemental material, sj-docx-1-anp-10.1177_00048674261461979 for Cardiometabolic screening, elevated results and clinical follow-up actions in Ma-ori and non-Ma-ori with psychosis in Canterbury, Aotearoa New Zealand by Nathan Monk, Emily Barrett, Julie Fitzjohn, Mau Te Rangimarie Clark, Melissa Kerdemelidis, Ruth Cunningham, Andre McLachlan, Waikaremoana Waitoki, Richard Porter and Cameron Lacey in Australian & New Zealand Journal of Psychiatry
