# Supplementary material for: Cardiometabolic screening, elevated results and clinical follow-up actions in Māori and non-Māori with psychosis in Canterbury, Aotearoa New Zealand
Source: Aust N Z J Psychiatry. 2026 Jul 4;60(10):931–40. doi: 10.1177/00048674261461979 (PMC13620362; doi:10.1177/00048674261461979)
Supplement: Supplementary file 1 [file sj-docx-1-anp-10.1177_00048674261461979.docx]

**Supplementary materials**

**Cardiometabolic screening, elevated results, and clinical follow-up actions in Māori and non-Māori with psychosis in Canterbury, Aotearoa New Zealand**

Nathan Monk, Emily Barrett, Julie Fitzjohn, Mau Te Rangimarie Clark, Melissa Kerdemelidis, Ruth Cunningham, Andre McLachlan, Waikaremoana Waitoki, Richard Porter, Cameron Lacey

*Australian and New Zealand Journal of Psychiatry*

Table S1 below summarises the diagnostic codes used for study inclusion.

| **Table S1**. Diagnostic codes for psychotic disorders | | |
| --- | --- | --- |
| Psychotic disorder | ICD-9/DSM-IV | ICD-10 |
| Schizophrenia | 29510-29569, 29580-29595 | F20 |
| Bipolar I | 29600-29602, 29604-29606, 29640-29642, 29644-29646, 29650-29652, 29654-29656, 29660-29662, 29664-29666, 2967, 29680, 29689 | F302-F309, F312, F313, F315, F316 |
| Schizoaffective | 29570-29575 | F25 |
| Depression with psychosis | 29624, 29634 | F323, F333 |
| Substance with psychosis | 2913-2915, 29211-29212 | F105, F115, F125, F135, F145, F155, F165, F175, F185, F195 |
| Other | 2988, 2971, 2973 | F22-F24, F28 |
| Organic | 29381, 29382 | F060, F062 |
| Not otherwise specified | 2989 | F29 |

| **Table S2**. Rates of lipids and HbA1c screening completion for Māori and non-Māori within 1 year of first specialist mental health service (SMHS) use in Canterbury between 1 August 2012 and 31 December 2019, models adjusted for rangatahi (16-24 years) vs. pakeke (25-64 years) sub-cohort | | | | | | | | | |
| --- | --- | --- | --- | --- | --- | --- | --- | --- | --- |
|  |  | Māori (*N* = 887) | |  |  | Non-Māori (*N* = 2953) | |  | OR (95% CI) |
| *All service users* | *n* | | % | | *n* | | % | |  |
| Lipids | 391 | | 44.1% | | 1276 | | 43.2% | | 1.02 (0.88, 1.19) |
| HbA1c | 292 | | 32.9% | | 918 | | 31.1% | | 1.08 (0.92, 1.27) |
|  |  | Māori (*N* = 440) | |  |  | Non-Māori (*N* = 1434) | |  | OR (95% CI) |
| *Olanzapine/clozapine users only* | *n* | | % | | *n* | | % | |  |
| Lipids | 240 | | 54.5% | | 734 | | 51.2% | | 1.10 (0.91, 1.33) |
| HbA1c | 193 | | 43.9% | | 541 | | 37.7% | | 1.22* (1.00, 1.48) |
| *Notes:* Both blood tests and antipsychotic use were recorded if they occurred within two months prior to or one year after each person’s study index date (so the follow-up period ended on 31 December 2020).  * *p* < .05; ** *p* < .01; *** *p* < .001 | | | | | | | | | |

| **Table S3**. Number of Māori and non-Māori Canterbury specialist mental health service (SMHS) users with an elevated lipids and HbA1c blood screening result between study entry and 31 December 2023, models adjusted for rangatahi (16-24 years) vs. pakeke (25-64 years) sub-cohort | | | | | | | | | | |
| --- | --- | --- | --- | --- | --- | --- | --- | --- | --- | --- |
|  |  | Māori (*N* = 887) | | |  |  | Non-Māori (*N* = 2953) | |  | OR (95% CI) |
| *All service users* | *n* | | % | *n* | | | | % | |  |
| Triglycerides >2.2 mmol/L (lipids) | 274 | | 30.9% | 681 | | | | 23.1% | | 1.55*** (1.31, 1.84) |
| TC:HDL-C >5 (lipids) | 202 | | 22.8% | 539 | | | | 18.3% | | 1.36*** (1.13, 1.64) |
| HbA1c >40 mmol/mol | 156 | | 17.6% | 343 | | | | 11.6% | | 1.81*** (1.46, 2.23) |
|  |  | Māori (*N* = 561) | | |  |  | Non-Māori (*N* = 1815) | |  | OR (95% CI) |
| *Any-time Olanzapine/clozapine users only* | *n* | | % | *n* | | | | % | |  |
| Triglycerides >2.2 mmol/L (lipids) | 223 | | 39.8% | 512 | | | | 28.2% | | 1.74*** (1.43, 2.13) |
| TC:HDL-C >5 (lipids) | 158 | | 28.2% | 393 | | | | 21.7% | | 1.45*** (1.16, 1.79) |
| HbA1c >40 mmol/mol | 112 | | 20.0% | 242 | | | | 13.3% | | 1.79*** (1.39, 2.30) |
| *Notes*: This analysis includes all blood tests taken after each person’s study index date (earliest possible date 1 August 2012, up to 31 December 2023).  Antipsychotic use for this analysis is recorded any time following study entry (and up to two months prior), so antipsychotic use is not necessarily contemporaneous with the elevated result.  * *p* < .05; ** *p* < .01; *** *p* < .001 | | | | | | | | | | |

| **Table S4**. Rate of follow-up action within 1 year following elevated lipids and/or HbA1c blood test results, Māori and non-Māori Canterbury specialist mental health service (SMHS) users with psychosis, models adjusted for rangatahi (16-24 years) vs. pakeke (25-64 years) sub-cohort | | | | | | | | |
| --- | --- | --- | --- | --- | --- | --- | --- | --- |
|  | Elevated result (*n*) | | % (*n*) re-tested following an elevated blood result | | | % (*n*) dispensed statin (lipids)/metformin (HbA1c) following elevated blood result | | |
|  | Māori  (*N* = 887) | Non-Māori  (*N* = 2953) | Māori | Non-Māori | OR (95% CI) | Māori | Non-Māori | OR (95% CI) |
| *All service users* |  |  |  |  |  |  |  |  |
| Triglycerides >2.2 mmol/L (lipids) | 274 | 681 | 38.0% (104) | 37.0% (252) | 1.03 (0.77, 1.38) | 16.1% (44) | 20.4% (139) | 0.85 (0.58, 1.24) |
| TC: HDL-C >5 (lipids) | 202 | 539 | 37.6% (76) | 38.4% (207) | 0.95 (0.68, 1.33) | 13.4% (27) | 16.0% (86) | 0.76 (0.43, 1.29) |
| HbA1c >40 mmol/mol | 156 | 343 | 51.9% (81) | 38.8% (133) | 1.64* (1.12, 2.42) | 42.9% (67) | 39.7% (136) | 1.13 (0.77, 1.67) |
|  | Māori  (*N* = 561) | Non-Māori  (*N* = 1815) | Māori | Non-Māori |  | Māori | Non-Māori |  |
| *Any-time olanzapine/clozapine users only* |  |  |  |  |  |  |  |  |
| Triglycerides >2.2 mmol/L (lipids) | 223 | 512 | 36.8% (86) | 37.9% (204) | 0.93 (0.67, 1.29) | 14.5% (32) | 19.1% (105) | 0.75 (0.48, 1.15) |
| TC: HDL-C >5 (lipids) | 158 | 393 | 38.0% (60) | 41.7% (164) | 0.85 (0.58, 1.25) | 14.3% (22) | 16.8% (66) | 0.71 (0.38, 1.28) |
| HbA1c >40 mmol/mol | 112 | 242 | 59.8% (67) | 42.6% (103) | 1.96** (1.24, 3.11) | 44.6% (50) | 37.6% (91) | 1.31 (0.83, 2.07) |
| *Notes*: All follow-up actions completed if recorded within 1 year of initial elevated blood result.  Follow-up percentages are the percentage of people with the corresponding elevated blood result.  Antipsychotic use for this analysis is recorded any time following study entry (and up to two months prior).  * *p* < .05; ** *p* < .01; *** *p* < .001 | | | | | | | | |
